# O‐GlcNAcylation regulates lysophosphatidic acid‐induced cell migration by regulating ERM family proteins

**DOI:** 10.1002/2211-5463.13404

**Published:** 2022-04-05

**Authors:** Minseok Song, Pann‐Ghill Suh

**Affiliations:** ^1^ Department of Life Sciences Yeungnam University Gyeongsan South Korea; ^2^ Korea Basic Science Research Institute (KBRI) Daegu South Korea

**Keywords:** ERM family proteins, lysophosphatidic acid, migration, O‐GlcNAcylation, ovarian cancer

## Abstract

O‐GlcNAcylation of intracellular proteins (O‐GlcNAc) is a post‐translational modification that often competes with phosphorylation in diverse cellular signaling pathways. Recent studies on human malignant tumors have demonstrated that O‐GlcNAc is implicated in cellular features relevant to metastasis. Here, we report that lysophosphatidic acid (LPA)‐induced ovarian cancer cell (OVCAR‐3) migration is regulated by O‐GlcNAc. We found that O‐GlcNAc modification of ERM family proteins, a membrane‐cytoskeletal crosslinker, was inversely correlated with its phosphorylation status. Moreover, the LPA‐induced formation of membrane protrusion structures, as well as the migration of OVCAR‐3 cells, was reduced by the accumulation of O‐GlcNAc. Collectively, these findings suggest that O‐GlcNAc is an essential signaling element controlling ERM family proteins involved in OVCAR‐3 cell migration.

## Graphical abstract

In this study, novel molecular mechanisms regulating ERM proteins, which play an important role in cell migration, were revealed. In addition to the regulation of activity by phosphorylation, o‐GlcNAc modification plays an important role in regulating the activity of ERM proteins involved in cell skeletal rearrangements and migration.

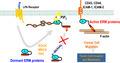

**Abbreviations**
LPA: lysophosphatidic acid
OGA: O‐GlcNAcylase
O‐GlcNAc: O‐GlcNAcylation
OGT: O‐GlcNAc Transferase
OVCAR‐3: ovarian cancer cell

O‐GlcNAcylation (O‐GlcNAc) is a dynamic post‐translational modification primarily localized in the intracellular compartment but not in the extracellular region [1, 2, 3, 4, 5, 6]. The number and types of proteins with these monosaccharide modifications are increasing. Transcription factors, tumor suppressor proteins, oncogene‐encoded proteins, RNA polymerase II, kinases and phosphatases, viral proteins, adapter proteins, nuclear pore proteins, and cytoskeletal proteins are reported to be modified with O‐GlcNAc [7, 8]. In terms of acting as a post‐translational modification, O‐GlcNAc is often compared with phosphorylation, which plays an essential role in the control of signaling pathways. [7, 8]. In some cases, the same amino acid residue is shared by both O‐GlcNAc and o‐phosphate. Therefore, by occupying the Ser/Thr site required for phosphorylation, O‐GlcNAc affects the phosphorylation pattern of major signaling molecules and, furthermore, the regulation of activity. O‐GlcNAc has an effect on enzymatic action, protein–protein interaction, subcellular localization, DNA‐transcriptional factor binding, and protein lifespan regulation.

Ezrin belongs to the ERM (ezrin/radixin/moesin) gene family [9, 10]. The ERM family is also included in the band 4.1 supergene family, which is composed of erythrocyte band 4.1, talin, protein tyrosine phosphatases, and a tumor suppressor gene, neurofibromatosis 2 [11, 12, 13, 14, 15, 16]. Like other proteins in the band 4.1 supergene family, ERM proteins are linker protein for membrane‐cytoskeleton interactions. In general, they are involved in diverse cellular processes, such as cell growth, motility, invasion, signal transduction, and cell–cell and cell–matrix recognition [17, 18, 19, 20, 21, 22, 23]. These functions play a very important role in the early development of tissues and organs, tumor progression, metastasis, and wound healing, [18, 24]. Various growth factors quickly induce membrane ruffling, ezrin phosphorylation, and translocation in certain types of cancer cells [25]. It is known that the expression level of ezrin is highly correlated with the invasive behavior of endometrial cancer cells [24]. Moreover, suppression of ezrin expression by small hairpin RNA could reduce metastasis in human breast cancer cells. [26]. For these reasons, ezrin is considered a key factor necessary for tumor metastasis.

ERM family proteins maintain a dormant state due to intramolecular interaction between the FERM and C‐terminal domains that cover the binding site of F‐actin. [27, 28, 29]. This intramolecular interaction is disrupted by phosphatidylinositol 4,5‐bisphosphate (PIP2) and phosphorylation of conserved threonine in the C‐terminal domain (T567) to expose F‐actin and membrane‐binding sites. Although the importance of phosphorylation in ERM family proteins is well characterized, the effect of other post‐translational modifications on ezrin was not investigated.

Here, we showed that ERM family proteins are modified by O‐GlcNAc in ovarian cancer cells. In addition, we found that LPA‐induced ERM phosphorylation and activation were impaired by increased O‐GlcNAc modification. Moreover, LPA‐induced translocation of ERM family proteins and ovarian cancer cell (OVCAR‐3) migration were diminished by increased O‐GlcNAc modification. We suggest that O‐GlcNAc plays important role in cancer cell migration by modulating ERM family proteins.

## Materials and methods

### Materials

Lipofectamine™ was purchased from Invitrogen (Carlsbad, CA, USA), and PUGNAc was from Toronto Research Chemicals (North York, Ontario, Canada). Cell culture accessories were obtained from BD Biosciences (Franklin Lakes, NJ, USA). FBS and Dulbecco's modified Eagle's medium (DMEM) were purchased from Gibco‐BRL and BioWhiteker (Walkersville, MD, USA), and the other Chemicals not mentioned were purchased from Sigma (St. Louis, MO, USA).

### Antibodies

CTD110.6 (Anti‐O‐GlcNAc) was provided by Dr. Gerald Hart (Johns Hopkins University, Baltimore, MD, USA). The α‐VSVG antibody was from New England Biolab (Ipswich, MA, USA), and the α‐phospho‐ERM antibody was purchased from Sigma. The α‐actin antibody and α‐phospho‐extracellular signal‐related kinase (ERK) antibody were obtained from ICN Biomedicals, Inc. (Aurora, OH, USA). Goat anti‐rabbit immunoglobulin (Ig) G conjugated with horseradish peroxidase and goat anti‐mouse IgA, IgM, and IgG were obtained from Kirkegaard & Perry Laboratories (Gaithersburg, MD, USA).

### Cell culture

Monolayer cultures of OVCAR‐3 cells (American Type Culture Collection, Manassas, VA, USA) were maintained in DMEM with 10% FBS. Each cell was grown at 37 °C in a humidified atmosphere containing 10% or 5% CO2.

### Plasmid construction and generation of adenovirus

Wild‐type and dominant‐negative ezrin constructs were kindly provided by Dr. Monique Arpin (Centre National de la Recherche Scientifique, Paris, France). The recombinant adenovirus, expressing the dominant‐negative form of ezrin, was produced in HEK‐293T cells, which were purified over CsCl gradients.

### Western blot analysis

OVCAR‐3 were lysed with hypotonic lysis buffer (5 mm Tris‐HCl, pH 7.4, 100 μm DTT, 50 μm EDTA, 50 mm NaF, 1 mm Na_3_VO_4_, and 1 mm PMSF) for western blot analysis. Twenty micrograms of lysates was separated on a 8% SDS‐polyacrylamide gel in denaturing condition and transferred to a nitrocellulose membrane. Membranes were incubated with 5% nonfat dry milk in phosphate‐buffered saline (PBS) containing 0.1% Triton X‐100. To detect O‐GlcNAc modification of cellular proteins, actin, VSVG‐tagged proteins, anti‐O‐GlcNAc, anti‐VSVG antibody, and anti‐actin antibody were incubated with the nitrocellulose membranes at 4 °C for overnight. Secondary antibodies conjugated to horseradish peroxidase were incubated at 1 : 10 000 dilution for the visualization with chemiluminescence.

### Pull‐down assay

A succinylated‐wheat germ agglutinin (sWGA) was used for pull‐down assays. The OVCAR‐3 cells were sonicated and centrifuged for 20 min at 10 000 × ***g***. Equal amounts of OVCAR‐3 cell extracts were subjected to the incubation with sWGA. The sWGA beads were rocked with lysates for 90 min at 4 °C. After completion of incubation, the sWGA beads were washed in wash buffer (50 mm HEPES, pH 7.4, 150 mm NaCl, and 1% Triton X‐100) more than three times. The proteins were eluted from sWGA beads with a 1× SDS sample buffer containing 50 mm DTT and 30 mm EDTA, loaded on 8% SDS/polyacrylamide gels, separated, transferred to nitrocellulose membranes, and visualized with standard western blot materials.

### Immunocytochemistry (ICC)

ICC was performed based on a previously reported procedure. Briefly, cells were plated and grown on PDL‐coated coverslips, rinsed three times with phosphate‐buffered saline (PBS), and then fixed with 4% (w/v) paraformaldehyde at 4 °C for 12 h. After rinsing two times with PBS, the coverslips were blocked with 1% goat serum and 0.1% Triton X‐100 at room temperature for 30 min. The coverslips were incubated with 2 μg·mL^−1^ anti‐phospho‐ERM or anti‐actin antibodies at room temperature for 2 h. After washing with PBS three times, goat anti‐rabbit antibody (TRITC‐labeled) and goat anti‐mouse antibody (FITC‐labeled) were incubated with the cells for 1 h. After washing with PBS three times, the slides were mounted for further analysis with confocal microscopy.

### Migration assay

A modified Boyden chamber assay was used to determine the degree of migration of OVCAR‐3 cells. 8‐μm pore‐containing migration chambers were coated with collagen (40 μL per each well, 20 μg·mL^−1^) for 2 h at 4 °C. After coating, wells were washed with 1× PBS two times. 2.5 × 10^4^ OVCAR‐3 cells were plated to the top chambers and then the chambers were transferred into the 96‐well plates, each well receiving indicated treatments. Next day, the non‐migrated cells were removed with a cotton swab from the top chamber. Successfully migrated OVCAR‐3 cells on the bottom of the chamber were fixed in 2% paraformaldehyde for 15 min and then permeabilized with 0.2% Triton X‐100 before Hoechst 33345 staining. The images of migrated cells were acquired with fluorescent microscopy at a 20‐fold magnification, and they were quantified by counting the Hoechst 33345 stained cells. At least three randomly selected fields were quantified for each experimental group.

## Results

### Modification of ezrin by O‐GlcNAc

**Fig. 1:**
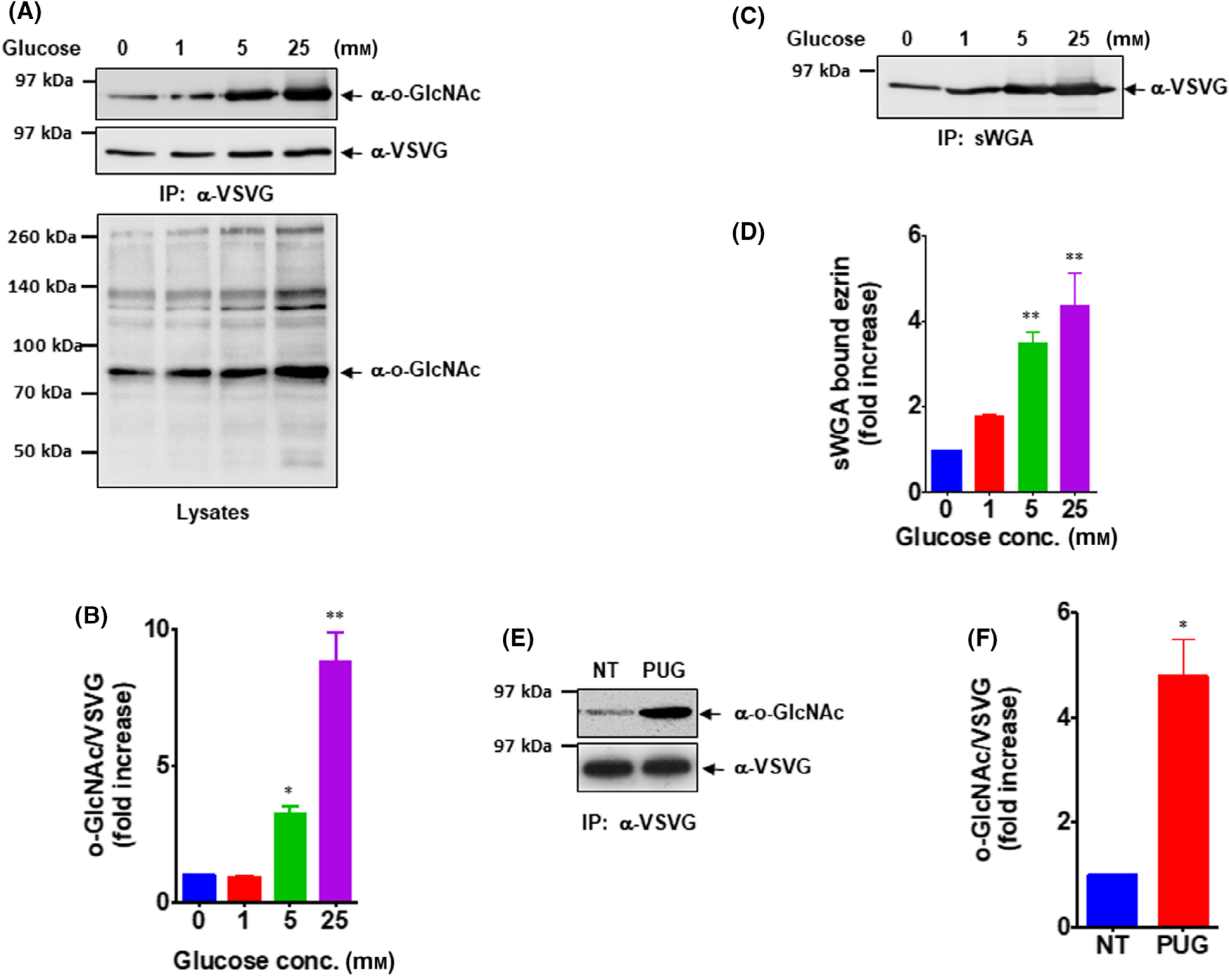
Ezrin is modified by O‐GlcNAc. (A) Ezrin was expressed in cells, and the indicated concentration of glucose was added to induce O‐GlcNAc modification. Then, cells were lysed, and ezrin was precipitated with VSVG antibody. The resulting immunoprecipitates were analyzed by western blot using an anti‐O‐GlcNAc antibody. Densitometric quantification of the results is shown in (B). Results are means ± SEM from 3 independent experiments. *P < 0.05, **P < 0.01 significantly different from control condition (one‐way ANOVA and Dunnett’s multiple comparisons test). (C) O‐GlcNAc modification of ezrin was verified by a pull‐down assay using O‐GlcNAc‐specific lectin, succinylated‐wheat germ agglutinin. The amount of precipitated ezrin was analyzed by western blot using an anti‐VSVG antibody. Densitometric quantification of the results is shown in (D). Results are means ± SEM from 3 independent experiments. **P < 0.01 significantly different from control condition (one‐way ANOVA and Dunnett’s multiple comparisons test). (E) PUGNAc, a specific inhibitor of O‐GlcNAcase, was used to accumulate O‐GlcNAc on ezrin. Then, cells were lysed, and ezrin was precipitated from the resulting lysates. The amount of O‐GlcNAc was analyzed using an anti‐O‐GlcNAc antibody. Densitometric quantification of the results is shown in (F). Results are means ± SEM from 3 independent experiments. *P < 0.05 significantly different from control condition (Student’s *t* test).

We performed a series of biochemical analyses to examine whether ezrin was modified by O‐GlcNAc. First, OVCAR‐3 cells expressing VSVG‐tagged ezrin were treated with the indicated concentration of glucose for 6 h to examine the cellular level of O‐GlcNAc modification (Fig. 1A). As shown in the lysate panel, O‐GlcNAc modification levels of total proteins gradually increased as the incubated glucose concentration increased. The O‐GlcNAc level of ezrin was probed with anti‐O‐GlcNAc antibodies after immunoprecipitation with anti‐VSVG antibodies. O‐GlcNAc modification of ezrin was barely detected in glucose‐starved or 1 mm glucose‐given conditions. However, it markedly increased with high concentrations of glucose. The O‐GlcNAc modification level of ezrin was 8.8‐fold higher in 25 mm glucose‐treated OVCAR‐3 cells compared with the glucose‐starved condition (Fig. 1B). To confirm the O‐GlcNAc modification of ezrin using an alternative method, the lysates of OVCAR‐3 cells from the indicated concentrations of glucose were subjected to a pull‐down assay using sWGA, a specific GlcNAc‐binding lectin. Consistent with the results shown with anti‐O‐GlcNAc antibodies, the amount of ezrin precipitated with sWGA was notably increased in high glucose conditions (Fig. 1C). Compared with the glucose‐starved condition, the amount of ezrin bound to sWGA was 4.3‐fold higher in 25 mm glucose‐treated OVCAR‐3 cells (Fig. 1D). The degree of O‐GlcNAc modification of target proteins is dynamically regulated by O‐GlcNAcase (OGA) and O‐GlcNAc transferase (OGT). Hence, we examined whether the O‐GlcNAc modification of ezrin was affected when OGA was inhibited. The results indicated that the O‐GlcNAc level of ezrin was markedly increased by treatment with PUGNAc, a specific inhibitor of OGA, in OVCAR‐3 cells (Fig. 1E,F). Collectively, these results demonstrate that ezrin is modified by O‐GlcNAc.

### Inhibition of ERM activation by the accumulation of O‐GlcNAc

**Fig. 2:**
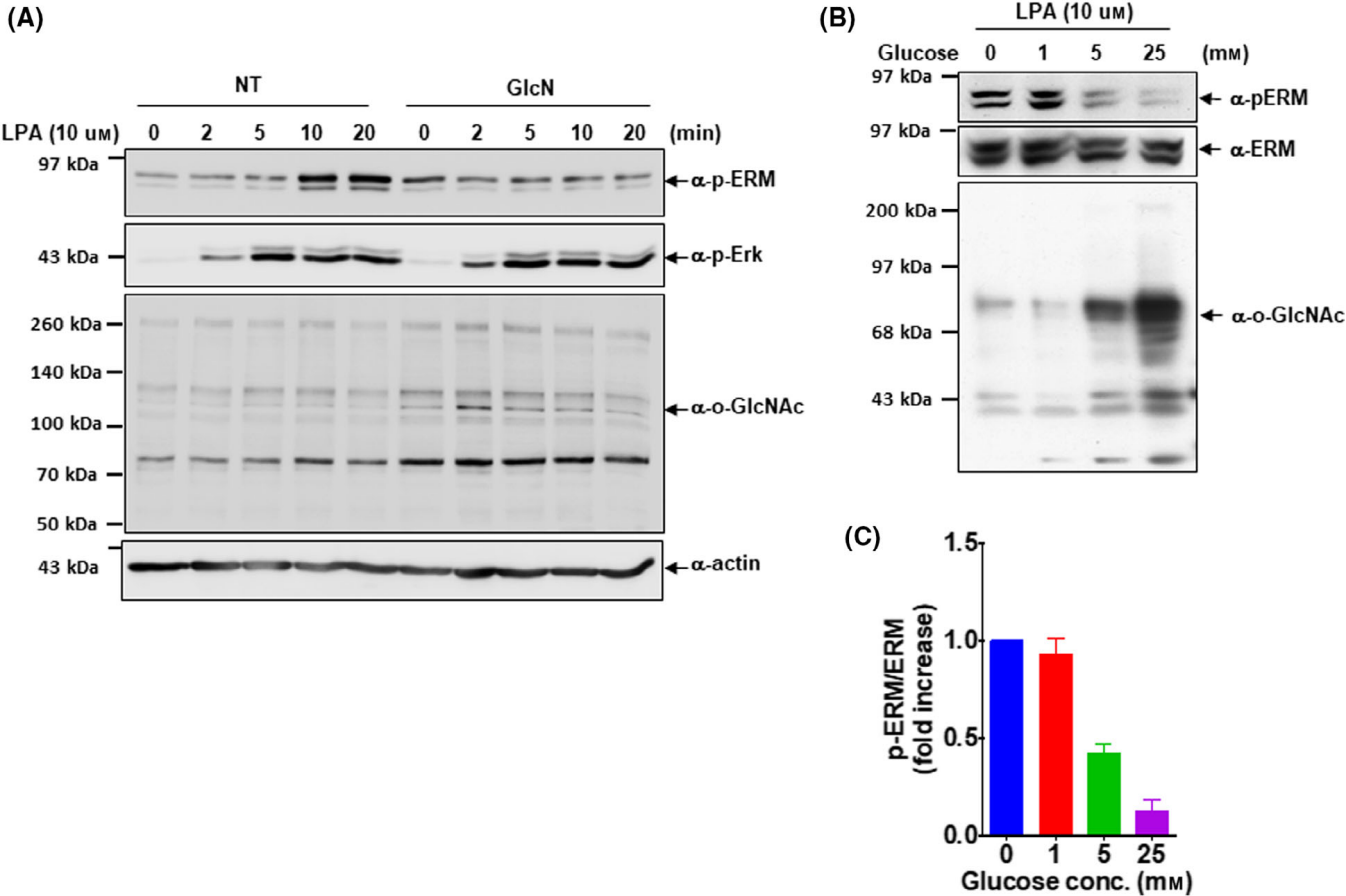
ERM (ezrin/radixin/moesin) phosphorylation is inversely correlated with O‐GlcNAc. (A) After glucose starvation for 2 h, cells were incubated with or without glucosamine for 2 h. Cells were then stimulated with 10 µm lysophosphatidic acid for the indicated time. The phosphorylation and O‐GlcNAc levels were analyzed by western blot using anti‐phospho‐ERM, anti‐phospho‐extracellular signal‐related kinase, and anti‐O‐GlcNAc antibody. (B) OVCAR‐3 cells were incubated with the indicated concentration of glucose to induce O‐GlcNAc modification. Cells were then stimulated with 10 µm lysophosphatidic acid for 10 min, and the lysates were analyzed by western blot. Densitometric quantification of the results is shown in (C). Results are means ± SEM from 3 independent experiments. **P < 0.01, ***P < 0.001 significantly different from control condition (one‐way ANOVA and Dunnett’s multiple comparisons test).

ERM family proteins are phosphorylated and activated by several kinases [27, 28, 29]. In ovarian cancer cells, lysophosphatidic acid (LPA) induces the phosphorylation and translocation of ERM family proteins [30, 31]. ERM activation is required for LPA‐induced cellular shape changes such as cytoskeletal remodeling and formation of membrane protrusions in OVCAR‐3 cells. As we found that ezrin was modified by O‐GlcNAc, we next investigated the effect of O‐GlcNAc modification on LPA‐induced phosphorylation and activation of ERM family proteins. OVCAR‐3 cells were incubated in the presence or absence of 3 M glucosamine for 2 h to raise the level of cellular O‐GlcNAc. Immunoblot analysis indicated that total cellular O‐GlcNAc levels markedly increased with glucosamine treatment (Fig. 2A). LPA stimulation induced Erk phosphorylation, showing a peak at 5 min in OVCAR‐3 cells. LPA‐induced Erk activation was not influenced by O‐GlcNAc accumulation with glucosamine treatment. LPA treatment induced phosphorylation of ERM proteins, showing a peak at 10 min; however, LPA‐induced ERM phosphorylation was remarkably reduced when cellular O‐GlcNAc modification was increased with glucosamine treatment (Fig. 2A). This result suggests that LPA‐induced ERM activation can be differentially regulated in response to cellular levels of O‐GlcNAc modification in OVCAR‐3 cells.

### Inverse correlation of ERM phosphorylation with O‐GlcNAc modification

Studies on the interplay between O‐GlcNAc and o‐phosphate have revealed the competitive and alternative occupancy of these modifications at the same or adjacent sites [32, 33]. As LPA‐induced ERM phosphorylation was diminished with the accumulation of O‐GlcNAc (Fig. 2A), we next investigated whether a mutually exclusive relationship between O‐GlcNAc and o‐phosphate modification on ERM family proteins exists. To this end, we tested how LPA‐induced ERM phosphorylation in OVCAR‐3 cells altered with the increasing dose of glucose in the culture medium. OVCAR‐3 cells were incubated with the indicated concentration of glucose for 6 h to increase O‐GlcNAc modification. As shown in the lysate panel, cellular O‐GlcNAc levels were potentiated by increasing the concentration of glucose. Intriguingly, immunoblot analysis demonstrated that LPA‐induced ERM phosphorylation was gradually reduced by increasing the concentration of glucose in the culture media (Fig. 2B, C). This result indicates that there is a competitive relationship between O‐GlcNAc and o‐phosphate on ERM family proteins in OVCAR‐3 cells.

### Impaired LPA‐induced translocation of ERM family proteins through the accumulation of O‐GlcNAc

**Fig. 3:**
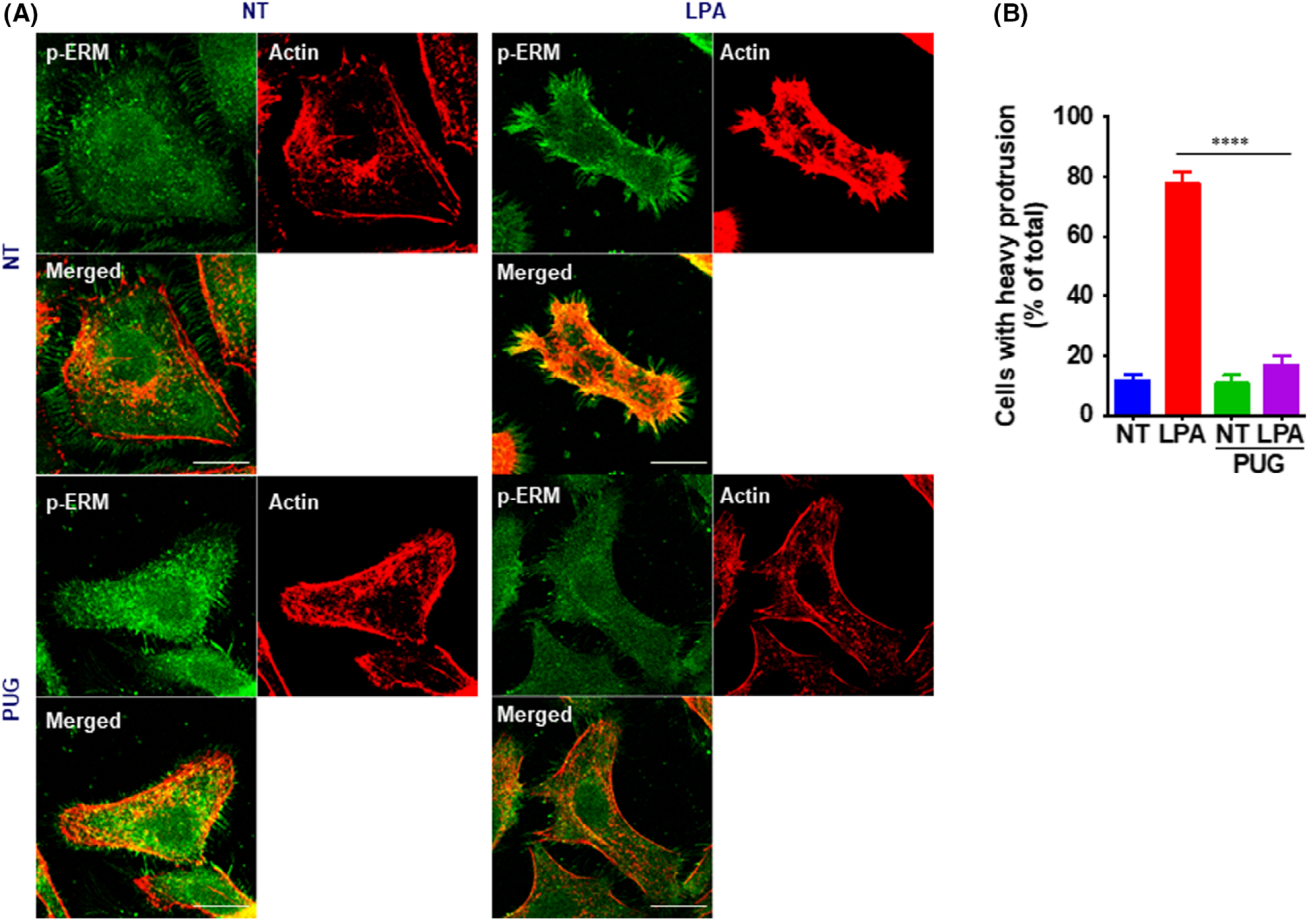
ERM (ezrin/radixin/moesin) activation is inhibited by the accumulation of O‐GlcNAc. (A) Cells were treated with or without PUGNAc, a specific inhibitor of O‐GlcNAcase, for 6 h to accumulate O‐GlcNAc modification. Cells were then stimulated with 10 µm lysophosphatidic acid for 10 min, and the localization of phospho‐ERM and actin was determined by immunofluorescence analysis. Scale bars, 20 μm (B) Images of each slide were acquired in 4–6 randomly selected high‐power fields (20× objective). A cell with a heavy protrusion was defined if it had a typical protrusion structure that covered more than 1/4 of the cell edge and quantified. Results are means ± SEM from 3 independent experiments. ****P < 0.0001 significantly different from control condition (Student’s *t* test).

It has been established that upon stimulation with LPA, ERM family proteins get phosphorylated, change conformation, and translocate to the cell surface to mediate membrane‐cytoskeletal interactions [27, 29]. We hypothesized that impaired LPA‐induced ERM phosphorylation in high O‐GlcNAc conditions might lead to the failure of ERM translocation to the cortical cytoskeleton. To determine whether O‐GlcNAc regulates ERM translocation, we investigated the intracellular distribution of phosphorylated ERM proteins and co‐localization with actin in cultured OVCAR‐3 cells using fluorescence microscopy. In untreated cells, phosphorylated ERM proteins were detected weakly and were mainly distributed in the cytoplasm, while actin was localized to the juxta‐membrane region (Fig. 3). Upon LPA stimulation, OVCAR‐3 cells developed filopodia, where phosphorylated ERM proteins were enriched and colocalized with the actin cytoskeleton. However, when PUGNAc was pretreated, OVCAR‐3 cells failed to develop a protrusion structure or increase phosphorylated ERM intensity. This result indicates that increased O‐GlcNAc impairs the activation and translocation of ERM family proteins in OVCAR‐3 cells.

### Inhibition of LPA‐induced migration by the accumulation of O‐GlcNAc in OVCAR‐3 cells

**Fig. 4:**
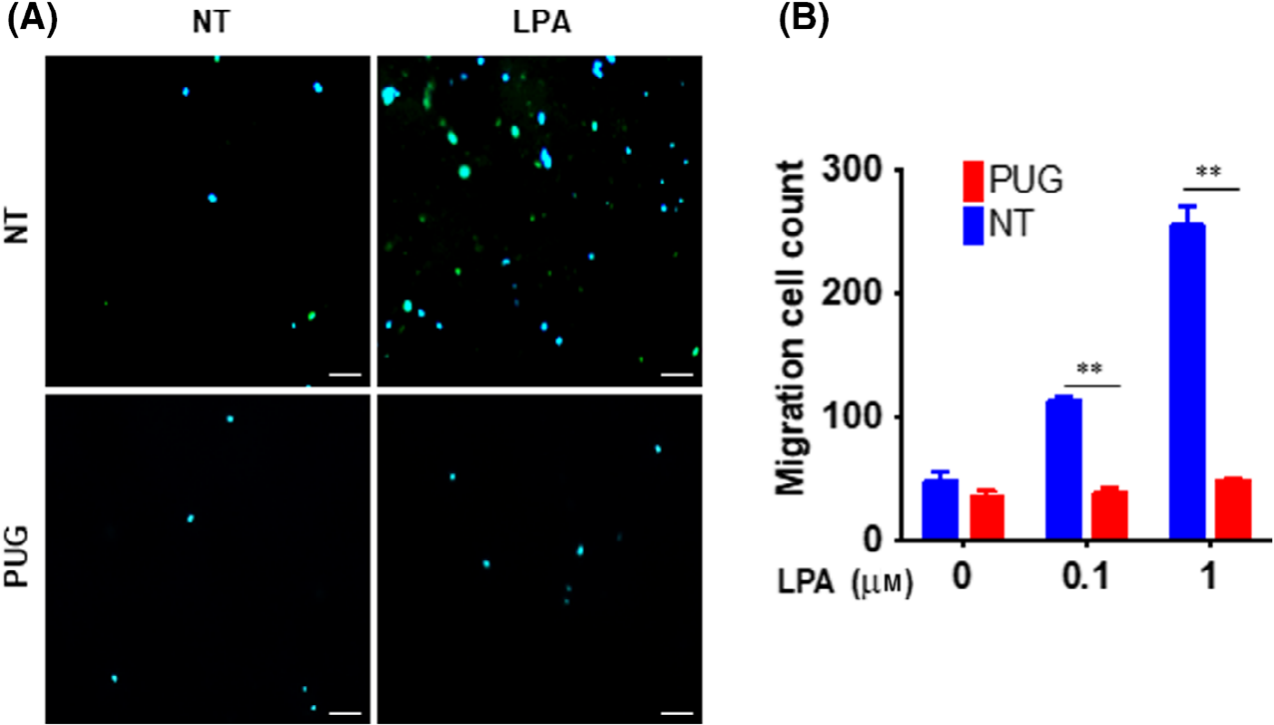
Lysophosphatidic acid‐induced migration is inhibited by the accumulation of O‐GlcNAc in ovarian cancer (OVCAR‐3) cells. (A) OVCAR‐3 cells were treated with or without PUGNAc for 4 h to induce O‐GlcNAc modification. The Boyden chamber assay was conducted using collagen‐coated 8‐μm pore‐containing membranes. Lysophosphatidic acid was added to the bottom chamber of the transwell, and cells were allowed to migrate for 3 h. (B) Migration was quantified by blind counting of the migrated cells on the lower surface of the membrane. At least three fields per chamber were counted using a 20× objective. Results are means ± SEM from 3 independent experiments. **P < 0.001 significantly different from control condition (Student's *t* test). Scale bars, 50 μm.

An increasing body of evidence suggests that ERM family proteins play an important role in cancer cell invasion and metastasis [18, 24, 34, 35, 36, 37]. Examination of ezrin levels in metastatic and non‐metastatic cells revealed that a significant increase in ezrin expression was generally observed in metastatic cells of various origins. As O‐GlcNAc modification of ERM family proteins inhibits the LPA‐induced phosphorylation and formation of filopodia structure, we next evaluated the effect of O‐GlcNAc on LPA‐induced OVCAR‐3 cell migration. ERM family proteins have been reported as an essential component of LPA‐induced migration of OVCAR‐3 cells. Indeed, LPA‐induced OVCAR‐3 cell migration was efficiently impaired by the dominant‐negative form of ezrin [31]. Hence, we performed a transwell *in vitro* migration assay to determine the effect of O‐GlcNAc accumulation with PUGNAc treatment on LPA‐induced OVCAR‐3 cell migration. As shown in Fig. 4A and B, LPA induced OVCAR‐3 cell migration potently in a dose‐dependent manner. However, pretreatment with PUGNAc impaired LPA‐induced cell migration almost completely. This result implies that O‐GlcNAc is an important inhibitory modification of LPA‐induced, ERM‐dependent ovarian cancer cell migration.

## Discussion

It is well established that the O‐GlcNAc modification site is similar in amino acid sequence to the phosphorylation site [6, 7, 8]. Moreover, in the case of c‐Myc [32], estrogen receptor β [38], SV‐40 large T antigen [39], and eNOS [40], O‐GlcNAc and o‐phosphate occupy the same site mutually exclusively. In this study, we confirmed the occurrence of O‐GlcNAc on ezrin in cells. We also found that the LPA‐induced phosphorylation of ERM family proteins was inhibited by increased O‐GlcNAc modification in cells. An earlier study suggested that Thr‐567 phosphorylation is essential for the functions of ezrin, such as actin tethering, protruding cell membrane, and cell migration [27, 28, 29]. We showed that increased O‐GlcNAc impairs the ERM‐mediated formation of membrane protrusion structure and OVCAR‐3 cell migration. Collectively, this study demonstrates that ERM function is modulated by O‐GlcNAc modification.

A study of primary human breast cancer demonstrated a decrease in overall O‐GlcNAc levels along with a significant increase in OGA enzyme activity. [41]. As O‐GlcNAc competes with o‐phosphate in many cases, the increase in OGA activity could shift the balance between these two modifications. Intracellular phosphorylation levels are tightly regulated by the cooperative action of protein kinases and protein phosphatases. Dysregulation of either of these activities can lead to cellular transformation. Therefore, the abnormal expression of OGA could evoke tumorigenesis by altering the regulation of protein O‐GlcNAc. This study strongly supports that as cells progress from normal to malignant, the regulatory mechanisms for protein O‐GlcNAc modification are disrupted, and the resulting reduction in O‐GlcNAc can lead to abnormal cell growth [41]. Currently, active research is ongoing on the role of O‐GlcNAc and O‐GlcNAc processing enzymes in various subjects such as cell growth, proliferation, and development of cancer.

ERM family proteins play a very important role in regulating cell morphology and cell migration. [17, 18, 19, 20, 21, 22, 23]. Activation of the ERM protein is caused by a conformational change induced by the interaction between the N‐terminal region of the FERM domain and PIP_2_ present in the plasma membrane. In addition, conserved threonine phosphorylation (ezrin‐T567, radixin‐T564, and moesin‐T558) in the C‐terminal domain of ERM family proteins contributes to the activation process. [27, 28, 29]. This sequential molecular event unmasks the intramolecular interaction between N terminus and C terminus. Here, we showed that ezrin, a member of the ERM family of proteins, is regulated by O‐GlcNAc modification. As LPA‐induced Erk phosphorylation was not influenced by accumulated O‐GlcNAc, the inhibition of LPA‐induced ERM phosphorylation by O‐GlcNAc is a specific event. O‐GlcNAc may strengthen the inhibitory intramolecular interaction in ERM family proteins. In addition, O‐GlcNAc could impair the interaction between ERM family proteins and activating kinase. However, the site of O‐GlcNAc modification on ERM family proteins has not yet been mapped. The site determination of O‐GlcNAc will provide a deeper understanding of the regulatory mechanisms of ERM family proteins.

Cancers usually result from somatic cell mutations that make or predispose a person to develop certain types of cancer. Interestingly, there is a growing awareness that signaling defects in nutrient metabolism can lead to abnormalities in cell growth and cancer. For example, loss‐of‐function mutations in tumor suppressor proteins such as LKB1 (AMPK signaling pathway) and TSC (mTOR signaling pathway) are associated with the pathogenesis of various types of cancer [42, 43]. O‐GlcNAc is transferred to the target protein by OGT using uridine diphosphate N‐acetylglucosamine, the end product of the hexosamine biosynthetic pathway. Dysregulated O‐GlcNAc modification has been commonly observed in various cancer cells, and OGT has been proposed as a novel therapeutic target for cancer treatment [44, 45]. Increased protein O‐GlcNAc modification and changes in the level of OGT or OGA expression are commonly observed features of many types of cancer, including leukemia, breast, prostate, and colon cancers [46]. However, decreased O‐GlcNAc modification has also been reported in tumor malignancy. In this study, we have shown that the hexosamine biosynthetic pathway, another important nutrient signaling pathway, modulates cancer cell migration by modulating ERM function with O‐GlcNAc modification.

In conclusion, our study demonstrates that increased O‐GlcNAc impairs the LPA‐induced migration of OVCAR‐3 cells, suggesting that a reduction in O‐GlcNAc levels and increased OGA expression might facilitate the migration of ovarian cancer cells. A growing body of evidence is showing that misregulation of O‐GlcNAc modification is implicated in various aspects of cancer progression. Determining precisely how O‐GlcNAc homeostasis is disturbed in the signaling pathway of cancer cell migration will be crucial for developing new therapeutic strategies to treat cancer metastasis.

## Conflict of interest

The authors declare that they have no conflicts of interest.

## Author contributions

MS and PS conceived and designed the project, MS acquired the data, MS analyzed and interpreted the data, and MS and PS wrote the paper.

## Data Availability

The data that support the findings of this study are available in the figures and the supporting information of this article.
